# Non-invasive prenatal testing to detect chromosome aneuploidies in 57,204 pregnancies

**DOI:** 10.1186/s13039-019-0441-5

**Published:** 2019-06-20

**Authors:** Ying Xue, Guodong Zhao, Hong Li, Qin Zhang, Jiafeng Lu, Bin Yu, Ting Wang

**Affiliations:** 1 The Affiliated Suzhou Hospital of Nanjing Medical University, Suzhou, 215000 Jiangsu Province China; 2 Suzhou Municipal Hospital, Suzhou, 215000 Jiangsu China; 3 Zhejiang University Kunshan Biotechnology Laboratory, Zhejiang University Kunshan Innovation Institute, Kunshan, 215300 Jiangsu China; 4 Changzhou Maternity and Child Health Care Hospital affiliated to Nanjing Medical University, Changzhou, Jiangsu Province, 213003 China

**Keywords:** NIPT, Chromosome aneuploidies, Next generation sequencing, Performance

## Abstract

**Background:**

Non-invasive prenatal testing (NIPT) has been widely used to detect common fetal chromosome aneuploidies, such as trisomy 13, 18, and 21 (T13, T18, and T21), and has expanded to sex chromosome aneuploidies (SCAs) during recent years, but few studies have reported NIPT detection of rare fetal chromosome aneuploidies (RCAs). In this study, we evaluated the clinical practical performance of NIPT to analyze all 24 chromosome aneuploidies among 57,204 pregnancies in the Suzhou area of China.

**Methods:**

This was a retrospective analysis of prospectively collected NIPT data from two next-generation sequencing (NGS) platforms (Illumina and Proton) obtained from The Affiliated Suzhou Hospital of Nanjing Medical University. NIPT results were validated by karyotyping or clinical follow-up.

**Results:**

NIPT using the Illumina platform identified 586 positive cases; fetal karyotyping and follow-up results validated 178 T21 cases, 49 T18 cases, 4 T13 cases, and 52 SCAs. On the Proton platform, 270 cases were positive during NIPT. Follow-up confirmed 85 T21 cases, 17 T18 cases, 4 T13 cases, 28 SCAs, and 1 fetal chromosome 22 aneuploidy case as true positives. There were 5 false-negative results, including 4 T21 and 1 T18 cases. The NGS platforms showed similar sensitivities and positive predictive values (PPVs) in detecting T21, T18, T13 and SCAs (*p* > 0.01). However, the Proton platform showed better specificity in detecting 45, X and the Illumina platform had better specificity in detecting T13 (*p* < 0.01). The major factor contributing to NIPT false-positives on the Illumina platform was false SCAs cases (65.11%). Maternal chromosome aneuploidies, maternal cancers, and confined placental mosaicism caused discordant results between fetal karyotyping and NIPT.

**Conclusion:**

NIPT with NGS showed good performance for detecting T13, T18, and T21. The Proton platform had better performance for detecting SCAs, but the NIPT accuracy rate for detecting RCAs was insufficient.

## Introduction

Since Lo et al. first discovered cell-free fetal DNA in the plasma of pregnant women in 1997 [1], next-generation sequencing (NGS)-based non-invasive prenatal testing (NIPT) for screening of fetal chromosome aneuploidies became reality [2]. Nowadays, NIPT has been widely used for detecting fetal chromosome trisomy 13, 18 and 21 (T13, T18, and T21) and sex chromosome aneuploidies (SCAs) with high sensitivity and specificity [3–5]. Rare fetal chromosome aneuploidies (RCAs) involve all fetal autosomal chromosomal abnormalities other than SCAs, T13, T18, and T21. However, most of the published data of NIPT have focused on three common aneuploidies (T13, T18, and T21) and SCAs [6]. The performance of NIPT for screening RCAs is still limited. Several recent reports revealed that RCAs also had great impact on prenatal diagnosis [7]. Some publications even revealed RCAs are less rare than previously thought and are often associated with poor obstetric outcomes [8]. Multiple groups have emphasized the importance of detecting RCAs for monitoring pregnancy health and minimizing the frequency of false-positive and false-negative NIPT results [9, 10].

With the development of NGS technologies, NIPT also has been applied in several sequencing platforms such as a semiconductor sequencing platform [11] and the Illumina sequencing platform [12]. With these advances, the U.S. Food & Drug Administration and Chinese Food & Drug Administration (CFDA) has approved NIPT to screen for common chromosomal aneuploidies. However, few studies have evaluated NIPT on different NGS platforms.

Here, we evaluated the performance of NIPT for detecting fetal T13, T18, and T21 and SCAs on Proton and Illumina sequencing platforms in pregnant women in the Suzhou area of China and examined the feasibility of using NIPT to screen for RCAs.

## Results

**Fig. 1:**
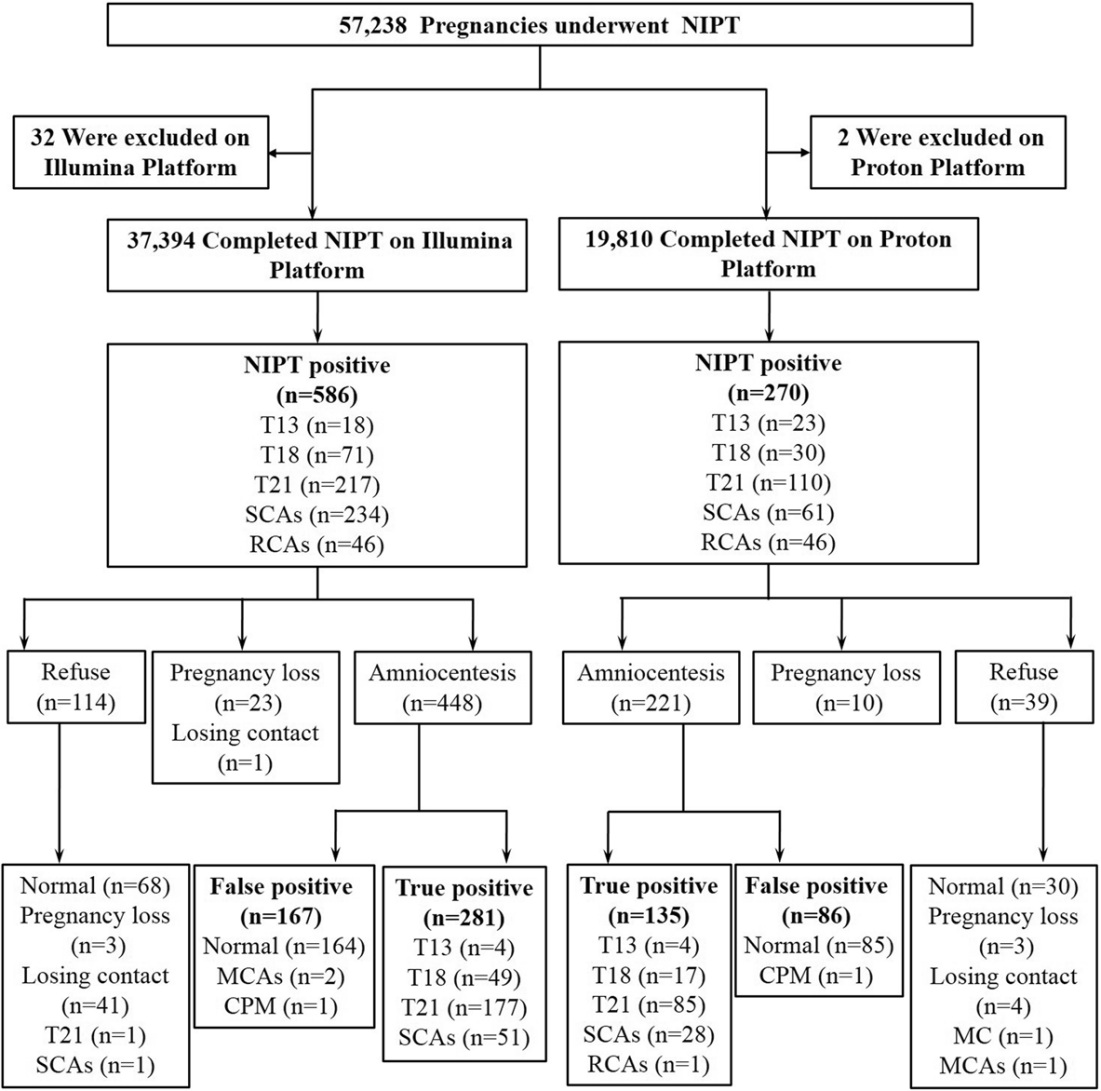
Study flow chart depicting the numbers of pregnancies that used NIPT for chromosome aneuploidy screening on two NGS platforms. CPM, confined placental mosaicism; MC, maternal cancer; MCAs, maternal chromosome aneuploidies; NGS, next-generations sequencing; RCA, rare chromosomal aneuploidy; SCA, sex chromosome aneuploidy; T, trisomy

Among 57,238 pregnancies who were undergo NIPT, 57,204 pregnancies completed NIPT: 37,394 using the Illumina platform and 19,810 on the Proton platform, and 34 subjects were excluded due to low fetal fraction (Fig. 1). The median gestational ages at the time of blood collection were 18.0 and 17.0 weeks for the Illumina and Proton platform, respectively. The median maternal ages for pregnancies on both NGS platforms were 30.0 years. Among the pregnant women detected on Illumina platform, 22.1% were advanced age pregnancies, 0.6% were twin pregnancies and 1.2% were IVF-ET pregnancies. While for pregnant women detected on Proton platform, 25.3% were advanced age pregnancies, 1.1% were twin pregnancies and 2.2% were IVF-ET pregnancies (Table 1).

**Table 1:** Distributions of maternal age and gestational age of pregnant women who underwent NIPT on two NGS platforms

	Illumina	Proton
MA (years)		
Median	30.0	30.0
Mean ± SD	30.1 ± 5.0	30.4 ± 5.0
Min-Max	16–50	15–49
AA (≥35)	8265 (22.1%)	5021 (25.3%)
AA with positive NIPT	158 (1.9%)	88 (1.8%)
GA (weeks)		
Median	18.0	17.0
Mean ± SD	17.7 ± 2.0	17.6 ± 2.1
Min-Max	11–30	11–30
Twin pregnancies	207 (0.6%)	218 (1.1%)
IVF-ET pregnancies	457 (1.2%)	429 (2.2%)

*AA* Advanced age, *GA* gestational age, *MA* maternal age, *NGS* next-generation sequencing

After NIPT, 586 (1.57%) pregnancies had positive results on the Illumina platform, including 18 for T13, 71 for T18, 217 for T21, 234 for SCAs, and 46 for RCAs. Among these, 448 (76.5%) cases underwent further prenatal diagnosis via amniocentesis; 218 fetal aneuploidies were confirmed, including 4 cases of T13, 49 cases of T18, 177 cases of T21, and 51 SCAs. For the 138 NIPT-positive cases that were not confirmed by fetal karyotyping, 114 cases refused confirmatory diagnosis, 23 cases ended with pregnancy loss, and 1 case was loss to follow-up. Among the 114 cases who decline invasive diagnostic testing, 68 cases had normal live births, three cases ended with pregnancy loss, 1 case had T21, 1 had an SCA, and 41 cases were loss to follow-up (Fig. 1).

On the Proton platform, 270 (1.36%) pregnancies had positive NIPT results, including 23 for T13, 30 for T18, 110 for T21, 61 for SCAs, and 46 for RCAs. Among these, 221 (81.9%) subjects consented to amniocentesis; 135 cases were confirmed true positive including 4 cases of T13, 17 cases of T18, 85 cases of T21, 28 SCAs, and 1 RCA. Of the 49 subjects with positive NIPT results that were not confirmed by fetal karyotyping, 39 declined further testing and 10 cases ended with pregnancy loss. Among the 39 cases who refused invasive diagnostic testing, 30 had normal live births, 3 cases ended with pregnancy loss, 4 were lost to follow-up, and 2 had normal live births but their mother had chromosomal abnormality or cancer (Fig. 1).

Among the 52 true-positive SCA results on the Illumina platform, 19 were 45, X, 10 cases were 47, XXX, 17 were 47, XXY, and the remaining 6 cases were 47, XYY. For the 28 cases with true-positive SCA results on the Proton platform, 4 cases were 45, X, 8 were 47, XXX, 12 were 47, XXY, and the remaining 4 were 47, XYY. With regard to the 92 cases with positive NIPT results for RCAs (46 each for the Illumina and Proton platforms), 43 cases (18 Illumina, 25 Proton) underwent prenatal diagnosis with amniocentesis, and only 1 case was confirmed as RCA (fetal chromosome 22 aneuploidy from Proton platform, Table 2).

**Table 2:** NIPT results for RCAs on two NGS platforms

RCAs	Illumina				Proton				Total			
	NIP	AM	TP	PPV (%)	NIP	AM	TP	PPV (%)	NIP	AM	TP	PPV (%)
7	15	5	0	0.00	10	4	0	0.00	25	9	0	0.00
2	6	1	0	0.00	5	3	0	0.00	11	4	0	0.00
16	4	2	/	0.00	3	2	0	0.00	7	4	/	0.00
3	2	1	0	0.00	6	4	0	0.00	8	5	0	0.00
22	1	1	0	0.00	7	3	1	33.33	8	4	1	25.00
8	2	/	/	/	4	3	0	0.00	6	3	0	0.00
5	1	1	0	0.00	3	2	0	0.00	4	3	0	0.00
10	3	1	0	0.00	1	/	/	/	4	1	0	0.00
14	3	2	0	0.00	1	1	0	0.00	4	3	0	0.00
20	1	1	0	0.00	3	2	0	0.00	4	3	0	0.00
1	2	2	0	0.00	/	/	/	/	2	2	0	0.00
9	1	/	/	/	1	/	/	/	2	/	/	/
11	2	1	0	0.00	/	/	/	/	2	1	0	0.00
15	2	/	/	/	/	/	/	/	2	/	/	/
19	1	/	/	/	1	/	/	/	2	/	/	/
4	/	/	/	/	1	1	0	/	1	/	/	/

*AM* amniocentesis, *NIP* NIPT positive, *PPV* positive predictive value, *RCA* rare chromosome aneuploidy, *TP* true positive

The sensitivities, specificities, and positive predictive values (PPVs) of NIPT using two NGS platforms for screening common chromosome aneuploidies and SCAs are shown in Table 3. Comparing performance between the two NGS platforms, there were no significant differences of sensitivity or PPV in detecting T21, T18, and T13, and there was no difference in specificity for detecting T21 or T18 (*p* > 0.01). However, the specificity of the Illumina platform in detecting T13 was significantly higher than that of Proton platform (*p* < 0.01).

**Table 3:** NIPT results for common fetal chromosome aneuploidies and SCAs on two NGS platforms

Chromosome aneuploidies		Illumina			Proton			*p*-value (Illumina vs Proton)		
		ST (%)	SP (%)	PPV (%)	ST (%)	SP (%)	PPV (%)	ST	SP	PPV
21		98.34	99.94	88.94	98.84	99.91	82.52	0.756	0.169	0.119
18		98.00	99.96	77.78	100.00	99.94	60.71	0.561	0.326	0.092
13		100.00	99.97	25.00	100.00	99.91	18.18	1.000	0.004	0.611
SCAs	45, X	100.00	99.79	19.39	100.00	99.95	28.57	1.000	< 0.001	0.426
	47, XXX	100.00	99.98	55.56	100.00	99.99	80.00	1.000	0.330	0.196
	47, XXY	100.00	99.97	55.17	100.00	99.99	92.31	1.000	0.031	0.018
	47, XYY	100.00	100.00	85.71	100.00	99.99	66.67	1.000	0.244	0.416

*NGS* next-generation sequencing, *PPV* positive predictive value, *SCA* sex chromosome aneuploidies, *SP* specificity, *ST* sensitivity

For SCA analysis, the sensitivities of NIPT for screening each SCA type on both NGS platforms were 100.00%. And the Proton platform had similar PPV in detecting SCAs compared with the Illumina platform (*p* > 0.01, Table 3). Regarding specificity analysis, the Proton platform showed significantly lower false positive rate to detect 45, X. Since most NIPT-positive RCA cases were confirmed as false positives, the PPVs for most RCAs (except fetal chromosome 22 aneuploidy) were 0% (Table 2).

The Illumina had 235 false-positive cases validated by fetal karyotyping and clinical follow-up, including 22 T21 cases, 16 T18 cases, 12 T13 cases, 153 SCAs, and 32 RCAs. Among the 118 false-positive cases identified with the Proton platform, 19 cases were T21, 12 were T18, 18 were T13, 28 were SCAs, and 39 cases were RCAs. Notably, the remaining six false-positives were due to maternal chromosome aneuploidies, confined placental mosaicism, or maternal malignancy (Table 4).

**Table 4:** NIPT false-positive cases caused by maternal chromosome aneuploidies, maternal cancer, and confined placental mosaicism

NGS platforms	NIPT results	Validated results
Illumina	45, X	CPM (45, X/46, XY)
	47, XXY	Maternal SCAs
	Chr1 aneuploidy	Maternal Chr1 aneuploidy
Proton	Chr7 aneuploidy	CPM (47, XX, + 7/46, XX)
	Chr8 aneuploidy	Maternal Chr8 aneuploidy
	Chr22 aneuploidy	Maternal malignancy

## Discussion

NIPT has been widely used for detecting common fetal chromosome aneuploidies such as T13, T18, and T21 and has even expanded to SCA detection. NIPT findings about RCAs also affect prenatal diagnoses, but few studies have reported these results. We evaluated the performances of two NGS platforms for detecting all 24 chromosome aneuploidies among 57,204 pregnancies that underwent NIPT in our clinical center. In 2015, Zhang et al. reported the overall sensitivity of NIPT on Illumina platform was 99.17% for T21, 98.24% for T18, and 100% for T13, and the specificity was 99.95% for T21, 99.95% for T18, and 99.96% for T13 [13]. Using the same approach, Zhou et al. showed the sensitivity and specificity of NIPT for detection of T21 and T18 and T13 were 100 and 99.9% [14]. While Francesco et al. revealed that 100% sensitivity and specificity using Proton platform for detection of T21 and T18 and T13 in a small group subjects [15]. And a system review summarized that the pooled sensitivities using different NGS platforms for T21 and T18 are 99.8% (95% CI 98.1–99.9%) and 97.7% (95% CI 95.8–98.7%) respectively, and the pooled sensitivity for T13 is 97.5% (95% CI 81.9–99.7%). The pooled specificity for all three trisomies is 99.9% (95 99.8–99.9%) [16]. In the present study, both Illumina and Proton platforms revealed high sensitivities and specificities for detection of T 21 and T18 and T13 (Table 3), which is comparable to previous studies. As for the PPV for detection of common fetal chromosome aneuploidies, we found similar PPVs for T21 and T18 compared to other studies, but the PPV for T13 was relatively lower in our population [14, 17]. This might due to the significantly lower incidence of T13 compared to T21 and T18, hence the number of T13 cases in each study was relatively low and led to PPV variation.

With regard to SCAs, we found that the Proton platform had better performance than the Illumina platform, which may be due to two reasons. Firstly, the Illumina NextSeq CN500 is based on a sequencing-by-synthesis principle, while the Proton platform uses a semiconductor sequencing technique. A previous study reported that the Illumina platform provides more accurate results than Ion Torrent Personal Genome Machine [18]. Sequencing depth and coverage are two key considerations for NGS, and greater sequencing depth and coverage always improve result accuracy [19]. In this study, the much longer reads and more uniquely mapped reads of the Proton platform narrow the gap between the two platforms, resulting in the similar sensitivities and PPVs in detecting T21, T18, T13 and SCAs, and even better specificity in detecting 45, X. Secondly, the number of SCAs in this study was insufficient, and the sample size of Illumina platform was almost double that for the Proton platform. Despite this, the results of the two platforms for SCA detection were consistent with reports in the literature [4, 20].

NIPT has been approved by the CFDA for detecting T13, T18, and T21, but it is not yet approved for SCA screening. This may be due to the high false-positive rates (65.11% for Illumina and 24.1% for Proton) that result in relatively lower specificity and unnecessary treatment (Table 3). It is important to consider that the prevalence of SCAs is 1:500, which is more common than the major trisomies [21]. The CFDA may soon consider NIPT for SCA detection based on more clinical data and improvement of NGS methods.

For RCA analysis, chromosome 7 aneuploidy accounted for the largest proportion of NIPT-positive cases on both platforms (Table 2). According to the previous studies, T7 is the most frequently detected chromosomal abnormality [7, 10], which was consistent with our results. However, only one case was confirmed as a true positive among 57,204 cases, and the most common reason for early abortion is the existence of a major fetal chromosomal abnormality [10]. The chromosome 22 aneuploidy case was detected on the Proton platform, perhaps due to the long reads and deeper sequencing leading to an unexpected harvest, but neither NGS platform is currently optimized for detecting RCAs. The follow-up period revealed several false-positive SCAs and RCAs cases caused by confined placental mosaicism, maternal chromosome aneuploidies, and maternal cancer (Table 4). This finding is consistent with several recent studies that reported discordant results between fetal karyotyping and NIPT [22, 23].

Nevertheless, there are several limitations of our study to be considered. First, due to different sequencing principles, the plasma volume, number of uniquely mapped reads and length of reads used in this study on two NGS platforms were also different, to achieve the best performance of each platform. Which might result in some imbalance of the comparison between two NGS platforms. Hence, we could adjust the procedures of two NGS platforms in the further study, using the same initial plasma volume, for example, to make a more accurate comparison between the two NGS platforms. Secondly, the subjects used in this study had median gestational weeks 17 to 18 weeks, the early pregnancy samples in this study only occupies a small portion. Which was due to the cost of NGS based prenatal testing is relative high, it still could not be a primary screening method for prenatal diagnosis in China. In the future, with the developing of sequencing method and the reduction of sequencing costs, we believe that NIPT can be used as a primary screening method for prenatal diagnosis, and it can cover the most subjects in the early pregnancy.

## Conclusion

In conclusion, both the Illumina and Proton platforms have good performance for detecting T13, T18, and T21; these NGS platforms can also be used for detecting SCAs, but the NIPT accuracy rate for detecting RCAs remains insufficient.

## Materials and methods

### Sample collection and sequencing

This was a retrospective study based on two NGS platforms (Illumina and Proton). NIPT data were collected from Center for Reproduction and Genetics at The Affiliated Suzhou Hospital of Nanjing Medical University. The Illumina platform data were from February 1, 2012 to December 31, 2017, and Proton platform data were collected between March 1, 2015 and December 31, 2017. The study was approved by the Institutional Review Board of The Affiliated Suzhou Hospital of Nanjing Medical University. All subjects provided written informed consent prior to participation.

#### Sequencing and data analysis on the proton platform

Ten milliliters of peripheral blood from each pregnant woman was drawn into a K_3_EDTA Vacuette tube (Becton-Dickinson, San Jose, CA, USA), and cfDNA from 600 μL of maternal plasma, was captured on magnetic beads, purified and concentrated, the final cfDNA was eluted in 35 μL elution buffer. And then 3 μL cfDNA was used for DNA concentration measurement and the remaining 32 μL cfDNA was used for the library construction following the manufacturer’s protocol (Suzhou Basecare Medical Device Co., Ltd., Suzhou, Jiangsu, China), and the sequencing library was loaded onto an Ion P1 chip. A standard 500-cycle of Ion torrent sequencing was run in a single-end sequencing model [24]. All sequencing data were mapped to the human reference genome of hg19 using bowtie2 software and four types of mapped reads (polymerase chain reaction duplicates, short reads < 35 bp, multi-mapped reads, and low-quality reads) were removed by a Perl script. The percentage of reads mapped to each chromosome was calculated using the number of uniquely mapped reads in a selected chromosome, divided by the count of uniquely mapped reads in all chromosomes (autosomal and sex) after normalizing the number of the uniquely mapping reads by LOESS regression to allow GC correction. Finally, approximately three million 125-bp uniquely mapping reads were generated, and chromosome z score values less than − 3.0 or greater than + 3.0 were classified as abnormal [24].

#### Sequencing and data analysis on the Illumina platform

Peripheral blood (10 ml) from each pregnant woman was drawn into a K_3_EDTA Vacuette tube (Becton-Dickinson) or a cell-free DNA storage tube (Cwbiotech, Taizhou, Jiangsu, China). Total cfDNA was extracted from 1.2 mL of plasma using a nucleic acid extraction kit from Berry Genomics Co., Limited following the product insert protocol. DNA was captured on magnetic beads, purified through washing steps and eluted in a final volume of 42 μL. And then quantity of DNA extracted was assessed using Qubit 3.0 fluorometer (Invitrogen, Life technologies). DNA libraries were constructed using 40.5 μL purified cfDNA following the manufacturer’s protocol (Berry Genomics Co., Limited, Beijng, China). Massively parallel sequencing was performed on the Illumina NextSeq CN500 platform [23]. For each sample, approximately three million 36-bp reads were generated, of which approximately two million were uniquely mapped to the hg19 reference genome. The fetal aneuploidy status was determined by Z-scores (normal range, − 3 < Z < 3) [25, 26].

### Chromosome karyotype analysis

Pregnant women with positive NIPT results for chromosome aneuploidies consented to invasive prenatal diagnosis. Amniocentesis was performed under sterile conditions and ultrasound guidance in our center. The amniocytes and peripheral blood cells were cultured at 37 °C. A total of 60 dividing phases were counted using an AI chromosome image analysis system based on the principle of “An International System for Human Cytogenetic Nomenclature, ISCN2013,” and 20 G-banded metaphases from each sample were analyzed in triplicate [27].

### Data analysis

For both Proton and Illumina sequencing platforms, a low fetal fraction of < 3% was reported as a result failure, and pregnant women with failure results after the initial blood sampling were followed up using a retest of a second blood sampling. Subjects were excluded if fetal fraction still lower than 3% at second testing.

Data were subjected to statistical analysis with IBM SPSS for Windows Version 22.0, and *t*-tests were used for comparisons between two samples at a significance level of *p* < 0.01. The sensitivity, specificity, and PPV of NIPT for detecting fetal chromosome aneuploidies were calculated.

## Data Availability

The data used to support the findings of this study are included within the article.

## Abbreviations

AA: Advanced age
AM: Amniocentesis
CFDA: Chinese Food & Drug Administration
CPM: Confined placental mosaicism
GA: Gestational age
IVF-ET: In vitro fertilization and embryo transfer
MA: Maternal age
MC: Maternal cancer
MCAs: Maternal chromosome aneuploidies
NGS: Next-generation sequencing
NIP: NIPT positive
NIPT: Non-invasive prenatal testinghas
PPV: Positive predictive value
RCAs: Rare fetal chromosome aneuploidies
SCAs: Sex chromosome aneuploidies
SP: Specificity
ST: Sensitivity
T13: Trisomy 13
T18: Trisomy 13
T21: Trisomy 21
TP: True positive
